# African swine fever virus transmission cycles in Central Europe: Evaluation of wild boar-soft tick contacts through detection of antibodies against *Ornithodoros erraticus* saliva antigen

**DOI:** 10.1186/s12917-015-0629-9

**Published:** 2016-01-04

**Authors:** Jana Pietschmann, Lina Mur, Sandra Blome, Martin Beer, Ricardo Pérez-Sánchez, Ana Oleaga, José Manuel Sánchez-Vizcaíno

**Affiliations:** Institute of Diagnostic Virology, Friedrich-Loeffler-Institut, Suedufer 10, 17493 Greifswald—Insel Riems, Germany; VISAVET Center and Animal Health Department, Veterinary School, Universidad Complutense de Madrid, Avenida Puerta de Hierro s/n, 28040 Madrid, Spain; Parasitología, IRNASA (CSIC), Cordel de Merinas, 40-52, 37008 Salamanca, Spain

**Keywords:** African swine fever, Transmission cycles, Wild boar, *Ornithodoros erraticus*, Tick saliva antigen, ELISA

## Abstract

**Background:**

African swine fever (ASF) is one of the most complex viral diseases affecting both domestic and wild pigs. It is caused by ASF virus (ASFV), the only DNA virus which can be efficiently transmitted by an arthropod vector, soft ticks of the genus *Ornithodoros*. These ticks can be part of ASFV-transmission cycles, and in Europe, *O. erraticus* was shown to be responsible for long-term maintenance of ASFV in Spain and Portugal. In 2014, the disease has been reintroduced into the European Union, affecting domestic pigs and, importantly, also the Eurasian wild boar population. In a first attempt to assess the risk of a tick-wild boar transmission cycle in Central Europe that would further complicate eradication of the disease, over 700 pre-existing serum samples from wild boar hunted in four representative German Federal States were investigated for the presence of antibodies directed against salivary antigen of *Ornithodoros erraticus* ticks using an indirect ELISA format.

**Results:**

Out of these samples, 16 reacted with moderate to high optical densities that could be indicative of tick bites in sampled wild boar. However, these samples did not show a spatial clustering (they were collected from distant geographical regions) and were of bad quality (hemolysis/impurities). Furthermore, all positive samples came from areas with suboptimal climate for soft ticks. For this reason, false positive reactions are likely.

**Conclusion:**

In conclusion, the study did not provide stringent evidence for soft tick-wild boar contact in the investigated German Federal States and thus, a relevant involvement in the epidemiology of ASF in German wild boar is unlikely. This fact would facilitate the eradication of ASF in the area, although other complex relations (wild boar biology and interactions with domestic pigs) need to be considered.

## Background

African swine fever (ASF) is one of the most important and complex notifiable diseases of both domestic and wild pigs. It is caused by the eponymous virus which belongs to the genus *Asfivirus* within the *Asfarviridae* family [1]. African swine fever virus (ASFV) is the only known DNA virus with an arthropod vector. The latter are soft ticks of the genus *Ornithodoros* [2]. In general, different transmission cycles are observed with ASFV: a sylvatic cycle, a soft tick-pig cycle, and a domestic cycle [3]. The former is of importance in Southern and Eastern Africa, where it involves warthogs and soft ticks of the *O. moubata* complex, while a similar sylvatic cycle in Europe, involving Eurasian wild boar and *O. erraticus* ticks has not been hitherto demonstrated [4]. The tick-pig cycle was up to now observed in Africa [5] and on the Iberian Peninsula [6], where it involved domestic pigs and *O. erraticus* ticks infesting the pig pens. This cycle can have a tremendous impact on transmission and long-term maintenance of ASF virus circulation [3] favoring endemic situations, especially in outdoor swine production [7]. However, once introduced into the domestic pig population, the virus does not rely on vector borne transmission, as both direct and indirect contacts are very efficient means of viral transmission [8].

The endophilous/ nidicolous ticks in the *O. erraticus* complex have been reported from the Iberian Peninsula, North and West Africa, and Western Asia [9–11]. On the Iberian Peninsula, *O. erraticus* ticks were found in close association with swine on free-range pig farms, hidden in holes, cracks and fissures inside and around pig-pens. They were also found in bird nests, in burrows of small mammals, under stones, or in the resting places of vertebrate host species, but always in the proximity of pig-pens [12]. So far, these soft ticks have never been reported from Central and Northern Europe [9, 11].

ASF was completely eradicated from Europe in the 1990’s except from the Italian island of Sardinia, which has been affected since 1978 [9]. In 2007, the disease was introduced into Georgia. Subsequently, it spread to neighboring Trans-Caucasian countries, the Russian Federation, and more recently (2014), it re-entered into the European Union affecting Lithuania, Poland, Latvia, and Estonia (OIE WAHID, visited March 23rd 2015). In most of the affected European countries, the disease was detected in domestic pigs of all production sectors and in wild boar. The involvement of the latter is of special importance as the very high density of wild boar in Central Europe could favor the establishment of endemic situations that are most difficult to control. Although no evidences were found in the past that wild boar were parasitized by Ornithodoros ticks [13], the absence of this relationship has not been clearly demonstrated by scientific data. Considering that the presence and involvement of soft ticks would certainly further complicate ASF eradication in the area [14], the assessment of a possible tick-wild boar cycle is urgently needed and would be required in the framework of legal binding ASF control within the European Union (Council Directive 2002/60/EC and Commission Decision 2003/422/EC).

In order to assess the possible role of soft ticks in Central Europe, over 700 serum samples from German wild boar were screened for antibodies against tick saliva antigen using an indirect ELISA previously developed and largely employed to screen domestic pigs in Spain. This ELISA uses a salivary gland extract (SGE) as antigen and provides 100 % specificity and sensitivity with experimentally infested pigs, decreasing to 90 % in field conditions [15–17].

## Methods

A total of 723 serum samples of hunted Eurasian wild boar from four different German Federal States, namely Rhineland-Palatinate (RP), North-Rhine Westphalia (NRW), Mecklenburg-Western Pomerania (MWP), and Brandenburg (BB), were investigated for antibodies against *O. erraticus* tick saliva antigen. The samples were obtained within the framework of an ongoing classical swine fever (CSF) surveillance program in the respective Federal States and were kindly provided by the competent regional veterinary laboratories (Rostock, Krefeld, Koblenz, and Frankfurt/Oder). The sampling areas were chosen to reflect the spatial extent of Germany (areas in the North-West, South-West, North-East, and East) and were taken from the sample collection at random.

The samples were transferred to the OIE-Reference Laboratory for ASF—Universidad Complutense de Madrid (UCM) to be tested in ELISA. All serum samples were analyzed against the SGE of *O. erraticus* and samples giving doubtful results were reanalyzed after deglycosylating the SGE with sodium metaperiodate to eliminate cross-reactivity by glyscosylated epitopes [Oleaga-Pérez et al., 1994]. The test shows an overall specificity of 100 % with experimental sera which drops to about 90 % under field conditions.

Sample results were normalized considering the controls of their plates (two positive controls and one negative control), and the sample to positive ratio (SP ratio, SP) was calculated for each sample. For the samples analyzed repeatedly in different plates, the maximum optical density (worst case scenario) was employed for the calculation of the final results.

## Results

**Fig. 1:**
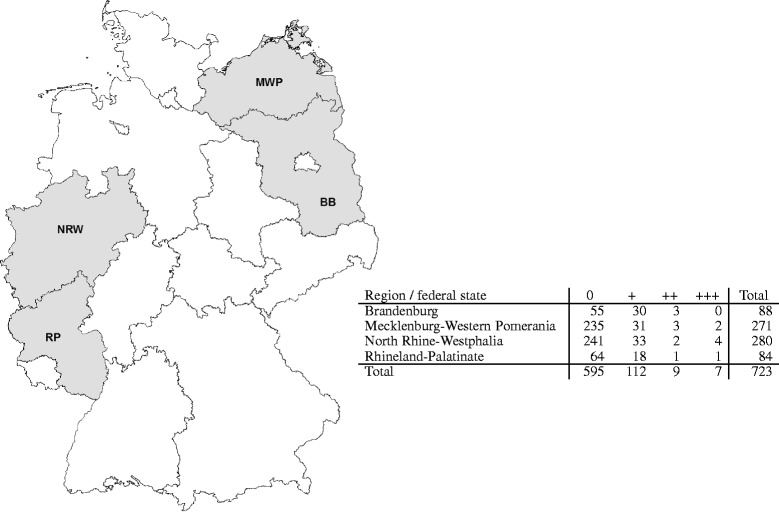
Origin of German wild boar sera analyzed utilizing the indirect antibody ELISA against salivary gland extract of O. erraticus ticks. Sampled areas are highlighted in grey and positive results are indicated by category in the adjacent table. Categorization of sera is done according to the sample to positive ratio (SP). The categories reflect the potential presence of Ornithodoros ticks in the respective regions from “negligible (0)” to “very likely (+++)” as defined in Table 1. NRW = North Rhine-Westphalia; RP = Rhineland-Palatinate; MWP = Mecklenburg-Western Pomarania; BB = Brandenburg. Copyright for the map by Geobasis-DE / BKG / GeoNutzV

Based on the established sample to positive (SP)-categorization (see Table 1), a total of 595 samples (82.3 % of all serum samples) were placed into category 0 (negligible risk for tick presence), 112 samples (15.5 % of sera) were assigned to category + (medium probability of tick presence) 9 (1.2 %) samples were categorized as ++ and seven samples (<1 %) were placed into the +++ category, which would mean, high and very high probability of tick presence, respectively. Of the seven samples in category +++, four samples originated from North-Rhine Westphalia (NRW), two from Mecklenburg-Western Pomerania (MWP), and one from Rhineland-Palatinate (RP). In category ++, samples were found from Brandenburg (BB; *n* = 3), MWP (*n* = 3), NRW (*n* = 2), and RP (*n* = 1). An overview of all SP results by region is presented in Fig. 1.

**Table 1:** Classification matrix according to the sample to positive ratio (SP)

SP	Category	Definition
<10	0	Negligible probability of tick presence
11–30	+	Medium probability of tick presence
31–50	++	High probability of tick presence
>50	+++	Very high probability of tick presence

Follow-up of positive samples placed in categories ++ and +++ revealed that these samples did not show spatial clustering and showed low sample quality with high degree of hemolysis and impurities. None of the samples originated from areas where the climate would suggest tick presence.

## Discussion

With the aim to assess the risk of a soft tick-wild boar transmission cycle of ASFV in Central Europe, and to explore the possible interest in direct tick sampling, more than 700 German wild boar sera available from an active classical swine fever surveillance were investigated by an indirect ELISA for the detection of antibodies against *Ornithodoros erraticus* tick saliva antigen. The results were categorized according to the SP ratio to indicate the possible presence of ticks in the region of origin.

From the total of 723 sera, 2.2 % were placed in categories that would indicate high and very high probability of tick presence. Given the fact that the ELISA has a specificity of 90 % under field conditions [15], these few high positive results could be within the false positive limits of the test system. This assumption is strengthened by the fact that these positive results were not spatially clustered, and were obtained from samples with low quality. Furthermore, these samples came from areas with suboptimal climate for tick maintenance, i.e., the respective soft ticks are highly resistant to high temperatures and dry conditions, but cold temperatures limit the development of their life cycle, which only starts when the external temperatures are in the range of 10–13 °C [18]. Therefore, the areas involved in the current study, presenting mean annual temperatures of 5 to 10 °C, do not seem to be optimal for *Ornithodoros* presence. Based on these facts, false positive reactions are quite likely. Those reactions are known to occur quite frequently in serological tests with samples of bad quality. Some ELISA tests for the detection of antibodies against ASFV also presented this problem [19], specifically when raw antigens were employed instead of recombinant or more purified antigens [20]. These results enhance the importance of obtaining good quality samples for a correct diagnosis, what is not always possible when working with materials from wild animals.

Apart from the above mentioned high suspect samples, another 15.5 % were placed in the category with medium probability of tick presence. This rate would definitively surpass the expected range of false positives (roughly 10 % under field conditions) and thus, the possibility that these sera and those classified as ++ and +++ are true positives cannot be completely ruled out. Only direct sampling of places where positive wild boar were hunted could give certainty. This would mean a tremendous effort in a region that is considered suboptimal for soft ticks and was never shown to harbor those species [9]. However, sampling attempts are under discussion in representative areas. Apart from true positive reactions, cross reactions with hard ticks have to be considered. While Pérez-Sánchez et al. [17] discarded cross reaction between *O. erraticus* and the most usual species of hard ticks that also parasitize swine on free-range pig farms in Southwestern Spain (namely, *Dermacentor marginatus*, *Hyalomma lusitanicum*, *R. sanguineus* and *R. bursa*), these authors did not include *Ixodes ricinus* in their study since this species is rarely found, if ever, in the holm-oak savannah lands where these free-range pig farms localized [21]. In contrast, *I. ricinus* is by far the most common species of tick in Germany and wild boar are among the hosts [22]. Thus, cross reaction between *O. erraticus* and *I. ricinus* should not be discarded and this could account for the positive results obtained with these sera.

## Conclusions

Based on the presented results, soft tick-wild boar contact and thus, a soft tick-wild boar transmission cycle of ASF in Germany seem unlikely. This conclusion has been previously suggested by other authors [13], but up to now, this is the first study supporting the previous hypothesis with field data.

These findings have an important impact on prevention and control strategies and should help to prioritize efforts and resources. Based on the presented data, the potential role of soft ticks in ASF persistence in German wild boar populations is mostly negligible and consequently, prevention measures should be focused on measures such as controlling artificial feeding and concentration points, analyzing home ranges and host interactions.

Further application of this serological test in other areas of Europe could help to understand and reveal the importance of ornithodoros ticks, and consequently assess its potential future implication in ASF epizootic cycles.

## References

[1] Takamatsu HD LK, Alonso C, Escribano JM, Martins C, Revilla Y, Salas ML (2011). Asfarviridae. Virus Taxonomy. Volume Ninth Report of the ICTV.

[2] Penrith ML, Vosloo W (2009). Review of African swine fever: transmission, spread and control. J S Afr Vet Assoc.

[3] Costard S, Mur L, Lubroth J, Sanchez-Vizcaino JM, Pfeiffer DU (2013). Epidemiology of African swine fever virus. Virus Res.

[4] Thomson GR (1985). The epidemiology of African swine fever: the role of free-living hosts in Africa. Onderstepoort J Vet Res.

[5] Plowright W, Parker J, Pierce MA (1969). The epidemiology of African swine fever in Africa. VetRec.

[6] Sanchez-Bojita A (1963). Reservorios del virus de la peste porcina Africana. Investigacion del virus de la P.P.A en los artropodos mediante la prueba de la hemoadsorcion. Bull Off Int Epizoot.

[7] Sánchez-Vizcaíno JM, Arias M, Zimmermann J, Karriker L, Ramirez A, Schwartz K (2012). African swine fever. Diseases of swine.

[8] Sanchez-Vizcaino JM, Mur L, Martinez-Lopez B (2012). African swine fever: an epidemiological update. Transbound Emerg Dis.

[9] Vial L (2009). Biological and ecological characteristics of soft ticks (Ixodida: Argasidae) and their impact for predicting tick and associated disease distribution. Parasite.

[10] Trape JF, Diatta G, Arnathau C, Bitam I, Sarih M, Belghyti D (2013). The epidemiology and geographic distribution of relapsing fever borreliosis in West and North Africa, with a review of the Ornithodoros erraticus complex (Acari: Ixodida). PLoS One.

[11] Boinas F, Ribeiro R, Madeira S, Palma M, de Carvalho I, Núncio S (2014). The medical and veterinary role of Ornithodoros erraticus complex ticks (Acari: Ixodida) on the Iberian Peninsula. J Vector Ecol.

[12] Oleaga-Perez A, Perez-Sanchez R, Encinas-Grandes A (1990). Distribution and biology of Ornithodoros erraticus in parts of Spain affected by African swine fever. Vet Rec.

[13] Scientific Opinion on African Swine Fever, EFSA Journal. 2010; 8(3):1556 [149 pp.].http://www.efsa.europa.eu/en/efsajournal/pub/155.

[14] Arias M, Sanchez-Vizcaino JM, Morilla A, Yoon KJ, Zimmerman JJ (2002). African Swine Fever Eradication: The Spanish Model. Trends in Emerging Viral Infections of Swine. Volume 1.

[15] Oleaga-Perez A, Perez-Sanchez R, Astigarraga A, Encinas-Grandes A (1994). Detection of pig farms with Ornithodoros erraticus by pig serology. Elimination of non-specific reactions by carbohydrate epitopes of salivary antigens. VetParasitol.

[16] Canals A, Oleaga A, Perez R, Dominguez J, Encinas A, Sanchez-Vizcaino JM (1990). Evaluation of an enzyme-linked immunosorbent assay to detect specific antibodies in pigs infested with the tick Ornithodoros erraticus (Argasidae). VetParasitol.

[17] Pérez-Sánchez R, Oleaga-Pérez A, Encinas-Grandes A (1992). Analysis of the specificity of the salivary antigens of Ornithodoros erraticus for the purpose of serological detection of swine farms harbouring the parasite. Parasite Immunol.

[18] Scientific report submitted to EFSA prepared by Sánchez-Vizcaíno, J.M., Martínez-López, B., Martínez-Avilés, M., Martins, C., Boinas, F., Vial, L., Michaud, V., Jori, F., Etter, E., Albina, E. and Roger, F. on African Swine Fever. (2009), 1–141.

[19] Gallardo C, Reis AL, Kalema-Zikusoka G, Malta J, Soler A, Blanco E (2009). Recombinant antigen targets for serodiagnosis of African swine fever. Clin Vaccine Immunol.

[20] Mur L, Boadella M, Martinez-Lopez B, Gallardo C, Gortazar C, Sanchez-Vizcaino JM (2012). Monitoring of African swine fever in the wild boar population of the most recent endemic area of Spain. Transbound Emerg Dis.

[21] Encinas Grandes A (1986). Ticks of the province of Salamanca (Central/NW Spain). Prevalence and parasitization intensity in dogs and domestic ungulates. Ann Parasitol Hum Comp.

[22] Schwarz A, Maier WA, Kistemann T, Kampen H (2009). Analysis of the distribution of the tick Ixodes ricinus L. (Acari: Ixodidae) in a nature reserve of western Germany using Geographic Information Systems. Int J Hyg Environ Health.

